# Size dependence of offspring production in isopods: a synthesis

**DOI:** 10.3897/zookeys.801.23677

**Published:** 2018-12-03

**Authors:** Andrzej Antoł, Marcin Czarnoleski

**Affiliations:** 1 Jagiellonian University, Institute of Environmental Sciences, Gronostajowa 7, 30-387 Kraków, Poland Jagiellonian University Kraków Poland

**Keywords:** clutch size, female size, indeterminate growth, life history evolution, offspring brooding, offspring size, parental care, trade-off

## Abstract

In isopods, parental care takes the form of offspring brooding in marsupial pouches. Marsupial brooding was an important step towards the origin of terrestrial lifestyles among isopods, but its potential role in shaping isopod life histories remains unknown. It is here considered that marsupial brooding imposes costs and creates a temporary association between the survival of mothers and that of their offspring. Integrating findings from different life history models, we predicted that the effects of marsupial brooding set selective conditions for the continuation of growth after maturation, which leads to indeterminate growth, and the production of larger offspring by larger females. Based on this perspective, a study on the size dependence of offspring production in the woodlouse *Porcellioscaber* was performed and the generality of the results was tested by reviewing the literature on offspring production in other isopods. In *P.scaber* and almost all the other studied isopods, clutch size is positively related to female size. Such dependence is a necessary pre-condition for the evolution of indeterminate growth. The body mass of *P.scaber* differed six-fold between the largest and smallest brooding females, indicating a high potential for post-maturation growth. Our review showed that offspring size is a rarely studied trait in isopods and that it correlates negatively with offspring number but positively with female size in nearly half of the studied species. Our study of *P.scaber* revealed similar patterns, but the positive effect of female size on offspring size occurred only in smaller broods, and the negative relation between clutch size and offspring size occurred only in larger females. We conclude that the intraspecific patterns of offspring production in isopods agree with theoretical predictions regarding the role of offspring brooding in shaping the adaptive patterns of female investment in growth, reproduction, and the parental care provided to individual offspring.

## Introduction

Most crustaceans engage in different types of parental care, which, in isopods, takes the form of offspring brooding in marsupia (Thiel 2000, Vogt 2016). During the moult preceding reproduction, the isopod female produces exoskeletal extrusions that form a marsupial pouch, which is used as a chamber for egg laying and carrying developing larvae (Hornung 2011, Appel et al. 2011). At the end of brooding, the female releases the offspring into the environment. In terrestrial species, individuals inside the marsupium undergo the change from the aqueous to the gaseous environment (Horváthová et al. 2015). Marsupial brooding was crucial for the origin of terrestrial lifestyles in isopods (Hornung 2011, Appel et al. 2011, Horváthová et al. 2017). Interestingly, land colonisation occurred independently at least twice in the evolutionary history of this group (Lins et al. 2017). Here, we consider that marsupial brooding plays a role in the evolution of life history strategies, especially by affecting adaptive patterns of female investment in growth, reproduction, and the parental care provided to individual offspring. To the best of our knowledge, this perspective remains largely unexplored in isopods.

The theory of life history evolution predicts that resource availability limits imposed by physiological and ecological circumstances forces organisms to optimise the lifetime allocation of investment among growth, reproduction and other competing demands to ensure the highest expected fitness under given mortality and production conditions (Stearns 1992). Adopting this basic principle, life history modelling has demonstrated that somatic growth is beneficial as long as one calorie invested in increasing body mass increases the future expected reproductive output by more than one calorie (Kozłowski 1992). Likewise, organisms are expected to optimise the amount of resources retained over unfavourable periods to fuel activities in favourable periods (Ejsmond et al. 2015); the timing of reproductive activity during a season, compromising the future prospects of offspring (Ejsmond et al. 2010); and the amount of resources invested in single offspring, compromising offspring number (Smith and Fretwell 1974). Developments in life history theory have led to an important conclusion: there is a wealth of distant optima with similar fitness consequences, which explains why life histories are so enormously diverse in nature (Stearns 1992, Czarnoleski et al. 2003, Kozłowski 2006).

A range of life history models predict the evolution of a bang-bang resource allocation strategy, which is associated with the complete cessation of growth after maturation and the so-called determinate growth pattern (Kozłowski and Wiegert 1987, Stearns 1992, Kozłowski 2006). In contrast, many isopods continue to moult after maturation, combining their capacity for reproduction with the capacity for somatic growth. This ability results in the potential for continuation of growth for the entire life span and the so-called indeterminate growth pattern. Beside isopods and some other crustaceans, indeterminate growth has evolved in annelids, molluscs, fish, amphibians, and reptiles (Kozłowski 1996). The prevalence of indeterminate growth in nature awaits explanation, but life history theory predicts that this growth strategy provides fitness advantages if the capacity to produce new tissue and/or survive strongly increases with body mass and if these capacities change discontinuously through time (Stearns 1992, Perrin and Sibly 1993, Kozłowski 2006). Modelling of optimal allocation has shown that discontinuities driven by either seasonal changes in mortality/productive capacity (Kozłowski and Teriokhin 1999) or unequal future prospects of offspring released into the environment at different times of the year (Ejsmond et al. 2010) lead to the evolution of alternating shifts between the investment in somatic growth and that in reproduction, resulting in the indeterminate growth pattern. For many organisms, including isopods, seasonality is the primary selective force responsible for the evolution of indeterminate growth. Nevertheless, specific characteristics of species biology, such as the reproduction via clutches instead of via a series of single offspring, can elicit discontinuous changes in mortality/production capacity, similar in principle to the effects of seasonality (Czarnoleski and Kozłowski 1998). Such characteristics can help explain why indeterminate growth originated among annuals or perennials living in non-seasonal environments. Heino and Kaitala (1996) designed a life history model for gill-brooding unionid mussels (e.g., *Sinanodontawoodiana*Labecka and Domagala 2018) and demonstrated that an indeterminate growth pattern can evolve in non-seasonal environments if carrying the offspring is associated with costs, either decreased physiological performance or increased mortality and with a temporary association between the fate of the offspring and the survival of the parent. Importantly, using a different model to explore the role of parental care in the evolution of offspring size among fish, Jørgensen et al. (2011) concluded that offspring brooding selects for the increased investment of larger females in individual offspring. For indeterminately growing animals, such a strategy involves constant changes in the optimal size of offspring as females increase their body mass. Under this strategy, the production of larger offspring is expected to require prolonged brooding, which temporarily links the fate of the offspring with that of the mother. If larger females have higher survival probability than do smaller females, then the increased investment in individual offspring becomes more beneficial for larger females. Overall, these theoretical considerations suggest that marsupial brooding might be an important driver of growth strategy and offspring size in isopods. To investigate this hypothesis, we performed a study on the common rough woodlouse (*Porcellioscaber*) and evaluated the generality of our results by analysing data from the literature on other isopods. We aimed at integrating information on intraspecific patterns of size dependence in offspring production over as wide a range of isopod species as possible. In particular, we focused on the relationships between female size and the number and size of offspring in broods and on evidence of an allocation trade-off between the number and size of the offspring in broods. Generally, we expected reproductive capacity to increase as females grow in size, which is the fundamental condition favouring the strategy of indeterminate growth (see above). Therefore, we expected a positive relationship between female body size and clutch mass/clutch size (hypothesis i). We also tested this relationship for non-linearity, assuming that a negative allometry would indicate an increased relative space limitation in larger females, whereas a positive allometry would indicate a decreased relative space limitation in larger females. Next, we examined whether the investment of females in individual offspring increased with the size of females, which should produce a positive correlation between the average offspring mass in a brood and female body mass (hypothesis ii). Finally, we analysed data on the mean mass of offspring in relation to the number of offspring per brood, looking for an allocation trade-off between offspring size and number (hypothesis iii).

## Materials and methods

### A case study of *Porcellioscaber*

In June–July 2014, individuals of *P.scaber* were collected in an old backyard in Kraków, Poland. In our study, we used females in the 3^rd^ and 4^th^ stages of brood development (classified according to Lardies et al. 2004a). Each gravid female was placed in a plastic box (100 ml). The boxes were perforated to provide aeration, lined with paper towel and supplied with a piece of moist sponge (water source), a piece of clay pot (shelter) and the dry leaves of the alder (*Alnusglutinosa*) and ash (*Fraxinusexcelsior*), which served as *ad libitum* food source. For additional control of humidity, the boxes were placed inside a larger plastic container with wet sand in the bottom. The container with boxes was placed in a shaded patio of the Institute of Environmental Sciences, Jagiellonian University in Kraków. Each day, the boxes were assessed for the presence of new offspring. Emerging offspring were collected, and the female was weighed to the nearest 0.001 mg (Mettler Toledo XP26, Greifensee, Switzerland). The clutches were dried for one hour at 60 °C in an oven (UFE 400, Memmert GmbH + Co. KG, Germany), and the dry mass of each clutch was measured to the nearest 0.001 mg (Mettler Toledo XP26, Greifensee). The offspring in each clutch were counted under a stereoscopic microscope. To calculate the mean dry mass of a single offspring, we divided the clutch dry mass by the number of offspring.

All statistical analyses were performed with R 3.4.1 software (R Core Team 2017), and the rgl package of R (Adler and Duncan Murdoch 2017) was used to create graphs. To test whether larger females produced heavier and larger clutches (hypothesis i) and larger offspring (hypothesis ii), we correlated clutch dry mass, clutch size, and mean offspring dry mass with female body mass. To evaluate the nature of these relations, we fitted linear and power regression models to our data and selected the best model using AIC. In this way, we did not a priori assume any particular relationship between the studied variables. When fitting our regression functions, we used either an ordinary least square (OLS) method or the weighted least square (WLS) method, which allowed us to account for the observed increase in the variance of dependent variables at higher values of an independent variable. Note that the OLS method assumes homogeneity in the variances of the independent variables. According to Knaub (2009), the issue of non-homogeneity can be overcome by using the WLS method, which assigns decreasing weights to observations with increasing levels of variance. Following Knaub’s (2009) procedure, we first ordered our data according to an increasing value of an independent variable to identify four quartiles. For data from the first quartile, the weights were calculated as an inverse of the highest value of the independent variable in this quartile (56.328 mg). For data from the other quartiles, the weights were calculated as the inverse of the actual value of the independent variable. To examine whether larger offspring emerged from smaller clutches (hypothesis iii), we used a multiple regression analysis with the mean offspring mass as a dependent variable and clutch size and female body mass as two independent variables. The use of a multiple regression allowed us to dissect the independent effects of each of the two independent variables. Thus, we also re-examined hypothesis (ii) regarding the link between female size and offspring size, with a control for the potential links between clutch size and offspring size. We allowed our model to consider an interaction between our two independent variables. Therefore, to assess the independent effects of each variable (partial regression), we estimated and tested this effect after centring the whole model in either the minimum or maximum value of each independent variable (Quinn and Keough 2002). The multiple regression analysis was performed with the use of either OLS or WLS, and the best model was chosen based on AIC.

### Intraspecific patterns in isopods

To evaluate the generality of our hypotheses (i–iii) and the empirical results for *P.scaber*, we reviewed the published literature on isopods for intraspecific information on at least one of the following relationships: clutch size with female size, offspring size with female size, and clutch size with offspring size. Relevant publications were identified by an extensive search of keywords in scientific databases, the review of reference lists of available publications and by personal communication with specialists in the field. Whenever we found relevant information regarding one of the three relationships, we classified the relationship as either statistically significant or non-significant; we also identified significant relationships as either positive or negative. If available, correlation coefficient (r) values were also assigned to each relationship. Traits used to study the relationship between female size and either clutch size or mass varied substantially among authors and species; therefore, we additionally recorded information regarding the types of measured traits. For each type of relationship, each species was classified according to the nature of this relationship, integrating all the results on a species reported in the literature. If a relationship for a given species was consistently reported to be significantly positive, significantly negative, or non-significant, the species was regarded as exhibiting a positive (+) or negative (-) relationship or no relationship (NS). Species for which mixed results were reported, showing either non-significant/significantly positive relationships or non-significant/significantly negative relationships were classified as NS/+ or NS/-, respectively. Ultimately, we used this integrated species information to calculate how frequently among the studied isopods a given pattern (+, -, NS, NS/+ and NS/-) of each relationship occurred. In addition, we used a 1-4 scale to evaluate the confidence in the support for each pattern (+, -, NS, NS/+ and NS/-) to predict the directions of the studied relationships (hypotheses i-iii). Consistently positive/negative relationships (+/-) were treated as providing reliable evidence to support or oppose a hypothesis. Non-significant patterns (NS) were regarded as not supporting a hypothesis, but we also considered the possibility that they might represent false negatives due to low statistical power. The level of support given by inconsistent results (NS/+ and NS/-) was dependent on the context. If among the non-significant and significant results, the significant results were consistent with our predictions, we treated the mixed results as weakly supporting our hypothesis. However, if the significant results were in conflict with the predictions, we regarded the mixed results as strongly opposing the hypothesis.

## Results

### A case study of *Porcellioscaber*

**Figure 1.**
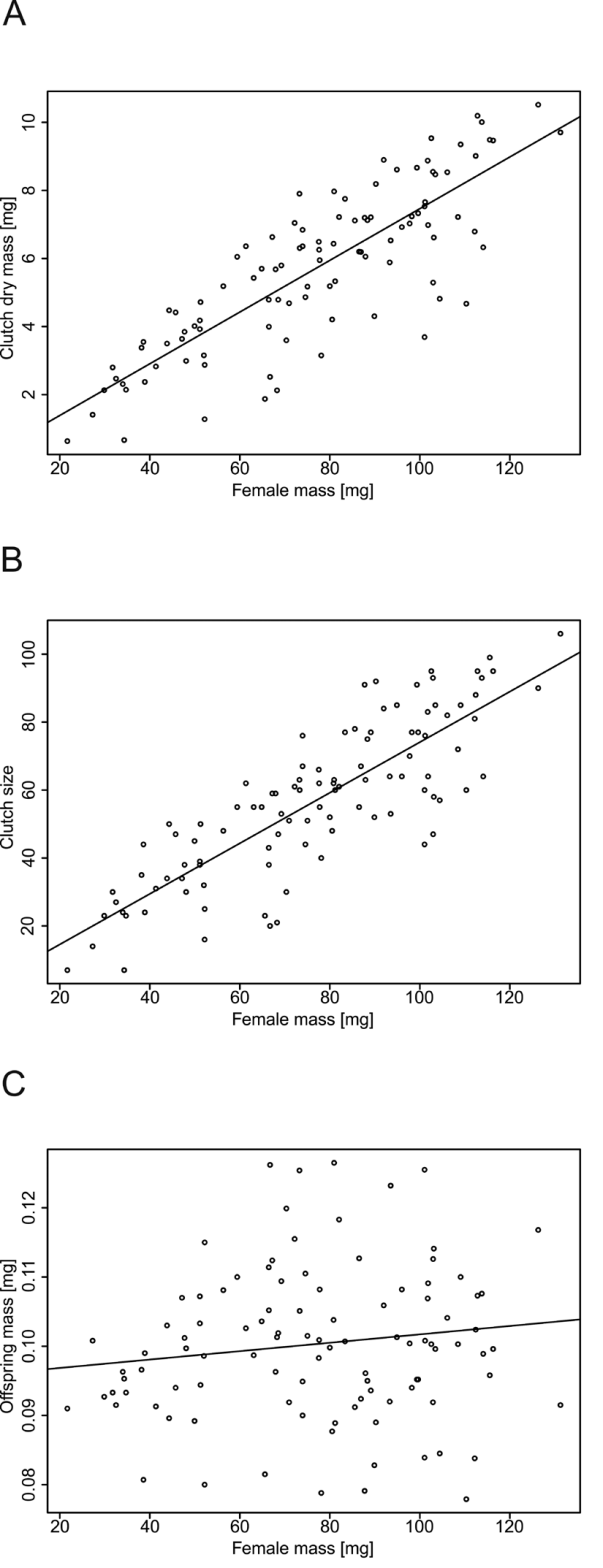
In *Porcellioscaber*, the dry mass of clutches (**A**) and clutch size (**B**) increased linearly with female body mass, but the mean dry mass of offspring did not depend on female mass in a consistent way (**C**). Lines represent fitted regressions **A** y = -0.13+0.08x (r = 0.83, p<0.001) **B** y = -0.32+0.74x (r = 0.83, p<0.001) **C** y = 0.1+0.00006x (r = 0.14, p = 0.15).

Among 101 brooding females of *P.scaber*, body mass ranged from 21.682 to 131.236 mg, clutch sizes ranged from 7 to 106 juveniles, and the mean dry body mass of offspring ranged from 0.078 to 0.126 mg between clutches. Larger females produced heavier (r = 0.83, t_1,99_ = 14.9, p<0.001, Fig. 1A) and larger clutches (r = 0.83, t_1,99_ = 15.09, p<0.001, Fig. 1B), but the mean offspring mass did not show a consistent relationship with female mass (r = 0.14, t_1,99_ = 1.44, p = 0.15, Fig. 1C). Comparison of AIC between the alternative regression models showed that a linear weighted regression produced the best fit to our data (Fig. 1). Therefore, we concluded that clutch size and clutch mass increased linearly with female body mass, which is consistent with our finding that the dry body mass of offspring did not change systematically with female body mass.

**Figure 2.**
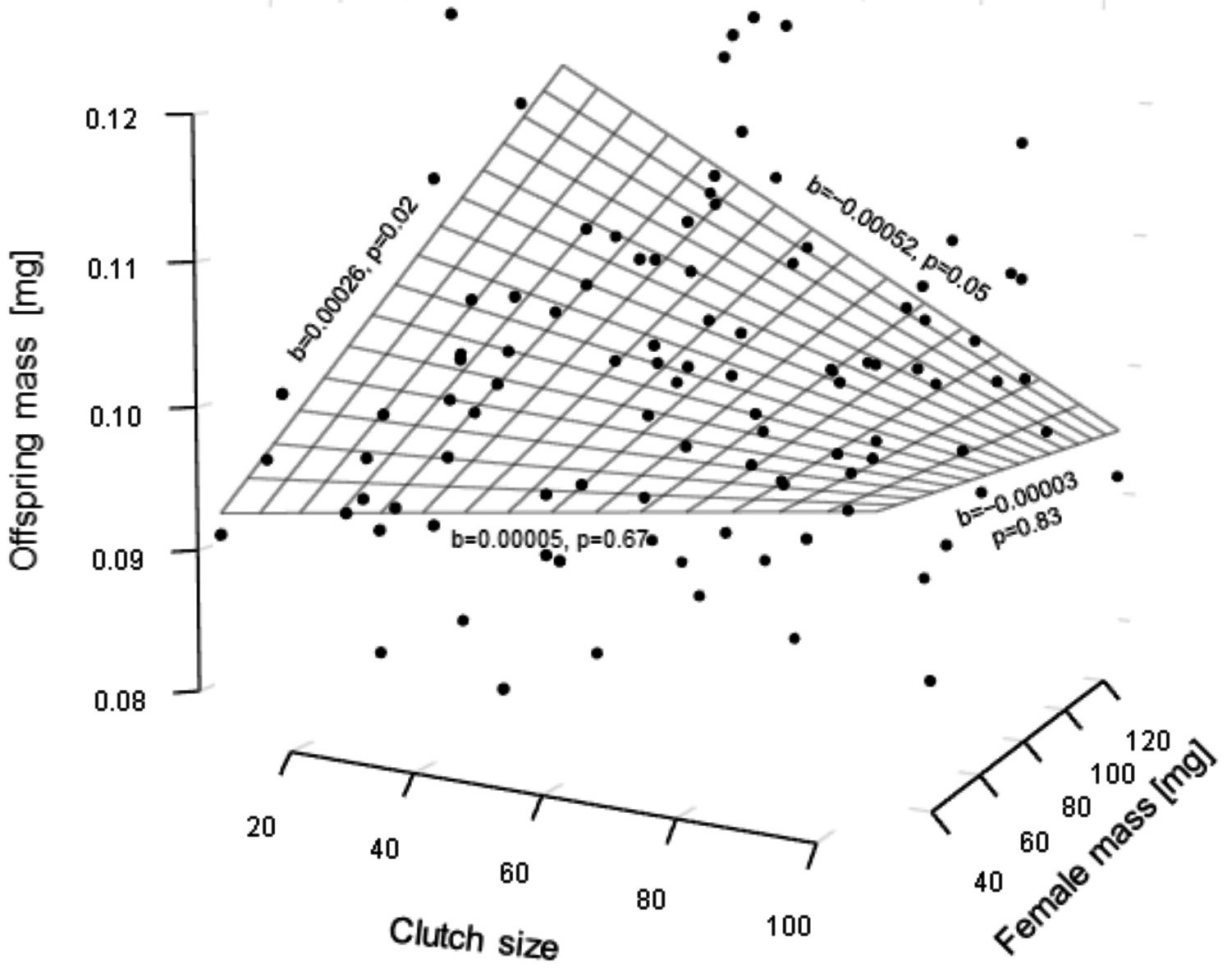
In *Porcellioscaber*, the heaviest offspring were released by large females that produced small clutches. The plane represents a multiple regression model fitted to the data; the partial slopes depicted on the edges were calculated by setting the other predictor value to its minimum and maximum values.

The results of the multiple regression analysis (Fig. 2) showed no effect of clutch size (t_1,97_ = 0.74, p = 0.46) and a positive effect of female mass (t_1,97_ = 2.38, p = 0.02) on the mean dry body mass of offspring. The interaction between the two independent variables was non-significant (t_1,97_ = -1.60, p = 0.11). When we centred the model at the value of the smallest broods (7 offspring), the positive link between offspring dry mass and female body mass was still significant (t_1,97_ = 2.39, p = 0.02), but the significance disappeared when we centred the model at the value of the largest clutches (107 offspring) (t_1,97_ = -0.22, p = 0.83). When we centred the model at the minimum female body mass (21.682 mg.), clutch size and offspring body mass appeared to be unrelated (t_1,97_=0.44, p=0.66), but centring at the maximum body mass (131.236 mg) revealed a negative relationship between clutch size and offspring body mass, though the effect was marginally significant (t_1,97_=-1.98, p=0.05). Overall, this analysis indicated that the largest offspring were produced by large females with small clutches.

### Intraspecific patterns in isopods

**Figure 3.**
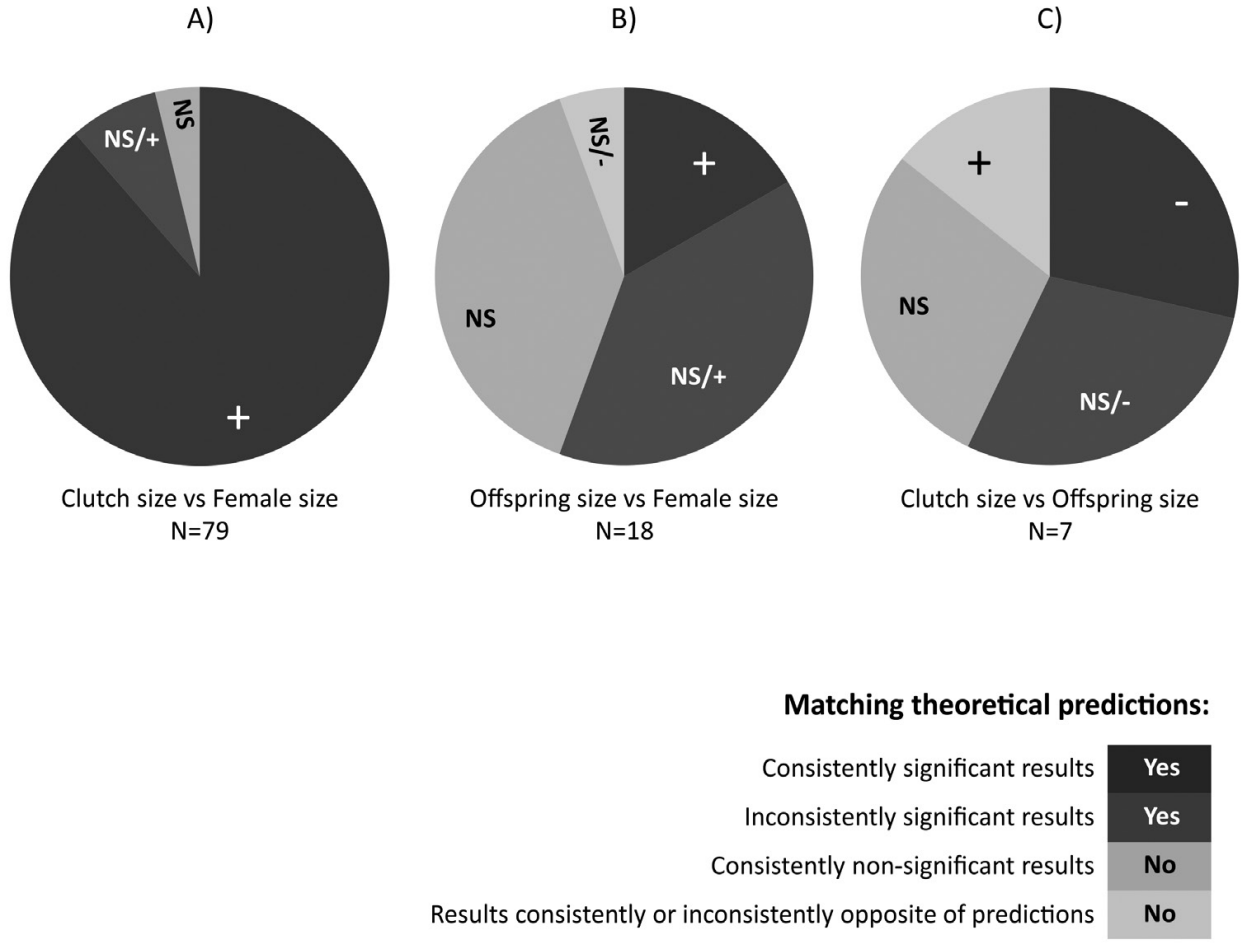
The literature search identified 79 species of isopods that were studied with respect to at least one of the following relationships: clutch size with female size (**A**), offspring size with female size (**B**), and clutch size with offspring size (**C**). Each graph shows how frequently a given nature of each relationship was found among the studied isopod species. The exact number of species for which the relationships **A, B, C** were evaluated is given by N. For each type of the relationships **A, B, C** each species was classified according to the nature of this relationship. If a relationship for a given species was consistently reported to be significantly positive, negative, or non-significant, the species was marked by a positive (+) or negative (-) symbol or by NS. Species for which mixed results were reported in the literature, showing either non-significant/significantly positive relationships or non-significant/significantly negative relationships, were marked by NS/+ or NS/-, respectively. Colour intensity indicates values along a 1–4 scale of confidence to the support provided by each relationship pattern (+, -, NS, NS/+ and NS/-) to hypotheses (i–iii). Relationship **A**: a positive relationship predicted between female body size and clutch mass/clutch size (hypothesis i). Relationship **B**: a positive correlation predicted between the average offspring mass in a brood and female body mass (hypothesis ii). Relationship **C**: a negative correlation predicted between the mean mass of offspring and the number of offspring per brood (hypothesis iii).

Our literature search identified a total of 79 species of isopods that were studied with respect to at least one of the following relationships: clutch size with female size (Fig. 3A), offspring size with female size (Fig. 3B), and clutch size with offspring size (Fig. 3C). Detailed results of the review are provided in Table 1S (Suppl. material 1). The effect of female size on clutch size was the most frequently studied relationship (79 species), while the relationships between female size and offspring size and between offspring size and clutch size were studied in only 18 and 7 species, respectively, including *P.scaber* as reported in this study. For the vast majority of the studied isopods (Fig. 3A), we found evidence that supports a positive relationship between female size and clutch size (hypothesis i). Importantly, we found no reports of the opposite pattern and only occasional reports of a non-significant pattern. However, the non-significant reports were typically found along with reports of significantly positive patterns, suggesting that many of the non-significant results might be false negatives. For nearly half of the species (Fig. 3B, C), we found evidence that supports a positive relationship between female size and offspring size (hypothesis ii) and a trade-off between offspring size and clutch size (hypothesis iii).

## Discussion

Growth patterns vary considerably in nature (Stearns 1992, Czarnoleski et al. 2003, 2005, Ejsmond et al. 2010), but understanding the origin of this variance is more challenging than it might initially appear. Our data on *P.scaber* suggest that this species of woodlouse has evolved a life history strategy with intense resource allocation to somatic growth in the reproductively mature stages. We found up to six-fold differences in body mass between the largest and the smallest brooding females, which suggests that only 20% of the body mass of a fully-grown female might be achieved before maturation, with the majority of growth potentially co-occurring with reproduction in such instances. Consistent with the idea that species with indeterminate growth should be characterized by a strong dependence of reproductive capacity on body size (hypothesis i), we found that larger females of *P.scaber* carried larger and heavier broods. This evidence clearly shows that mature females can gain reproductive capacity by further increasing body mass. The results of our literature search indicate that such size dependence is widespread among other isopod species. Interestingly, we found no reports of a negative pattern of this relationship and few reports of non-significant effects of female size on clutch size, which are likely to be false negatives. A strong size dependence of reproductive capacity promotes the evolution of iteroparous breeding with indeterminate growth, but alone, it is not sufficient to explain such evolution (Heino and Kaitala 1996, Czarnoleski and Kozłowski 1998). In fact, some isopods, such as *Ligiaoceanica*, have evolved a semelparous breeding strategy with determinate growth, despite the size dependence of reproductive capacity (Sutton et al. 1984, Willows 1987). Given this, what might be the ultimate drivers of the evolution of indeterminate growth in isopods? A life history theory calls attention to the pattern of resource allocation among growth, reproduction and other competing demands, which should be optimised to ensure the highest expected lifetime fitness in given mortality and production conditions (Stearns 1992, Kozłowski 2006). Considering this idea, the alternating allocations between growth and reproduction that lead to indeterminate growth reflect changes in allocation optima, with temporal shifts in the capacity to survive and/or reproduce. The woodlouse *P.scaber* and many other isopod species inhabit seasonal environments, and life history models have demonstrated that seasonal alternations of the periods suitable for survival, offspring production, and growth with less favourable periods establish the selective forces that favour the continuation of somatic growth after maturation (Kozłowski and Teriokhin 1996, Czarnoleski and Kozłowski 1998, Ejsmond et al. 2010). However, as suggested by Heino and Kaitala (1996) and Czarnoleski and Kozłowski (1998), the strategy of indeterminate growth might also bring additional fitness benefits if organisms engage in offspring brooding. Carrying offspring creates temporary changes in mortality/physiological performance and links between the fate of the offspring and that of the mother, leading to shifts in the optimality of growth and reproduction through time. Unfortunately, the costs associated with offspring brooding are poorly studied in isopods, but we might expect them in the form of increased vulnerability to predation and/or increased energetic costs associated with locomotion and supplementation of offspring. For example, Kight and Ozga (2001) observed that gravid females of *Porcelliolaevis* were less mobile than were non-gravid females. In addition, female isopods are postulated to regulate the pH and osmolality of their marsupial fluids and provision their broods with necessary resources via the so-called cotyledon (Lardies et al. 2004a). Furthermore, Lardies et al. (2004a) showed that gravid females had lower ingestion rates and digestibility and higher metabolic rates than did non-gravid ones. Interestingly, Perrin and Sibly (1993) suggested another mechanism that favours indeterminate growth among offspring brooders, which is non-exclusive of the hypothesis of a role of discontinuities in mortality/physiological capacity. If current offspring production is limited by the space provided by the brooding cavities rather than by the physiological capacity to produce new tissue, organisms are selected to direct surplus resources to further somatic growth, thereby increasing their fertility at the following reproductive event. There is some evidence to suggest that the maximal reproductive performance of isopods might be restricted by the volume of the marsupial pouches (Lardies et al. 2004a, Appel et al. 2011). Nevertheless, we found no indication that such limitations change with body size in females of *P.scaber*: The relationship between clutch size and female size did not deviate from linearity. In addition, we detected substantial variance in the mass of clutches produced by females of a given body size, which suggests that reproductive capacity might not be entirely dependent on the space limitation of the marsupium, unless the volume of the marsupium is highly variable at a given body mass.

Our data on *P.scaber* show that the dry body mass of offspring differed between broods by as much as 62%. A significant part of this variance was linked to differences in clutch size and female body mass, but the pattern of this dependence was complex. Supporting hypothesis ii, the size of offspring was positively related to female size, but this pattern existed only if we considered small clutches. Focusing on larger clutches, we found no apparent relationship between offspring size and female size. In accord with hypothesis iii, the size and number of offspring were inversely related, but this pattern existed only among larger females. In broods produced by smaller females, the two traits were not correlated. To date, studies of isopods have only occasionally addressed the question of whether offspring size changes with either female size or clutch size. According to our literature search, the relationships between female size and offspring size and between offspring size and clutch size have only been studied in 18 and 7 species, respectively. For nearly half of these species, we found evidence that supports a positive relationship between female size and offspring size (hypothesis ii) and a trade-off between offspring size and clutch size (hypothesis iii). It is suggestive that all studies that failed to find evidence of such a trade-off (Fig. 3C) overlooked the potential effects of female size in the statistical analysis of offspring size and clutch size data. In effect, many of these results might represent false negatives because differences in clutch size driven by female size are not primarily generated by the trade-off between offspring size and number but rather by the higher capacity of larger animals to produce new tissue (as shown in Figs 1A, 3A). Furthermore, the positive effects of female size on offspring size can lead to a positive correlation between offspring size and clutch size. Apparently, this is the case in the isopod *Bethaluspretoriensis* (Telford and Dangerfield 1995), which was the only species we found for which a positive association between clutch size and offspring size was reported; furthermore, a positive association between offspring size and female size was found in this species (see Suppl. material 1).

Examples of life history strategies in which offspring size is a function of parent size are rare in nature, and their evolutionary origins are puzzling (Rollinson and Rowe 2016). Apart from isopods, positive relationships between offspring size and female size have previously been reported in some other arthropods (Fox and Czesak 2000) and some species of snakes (Ford and Seigel 2011) and fish (Hendry et al. 2001, Hendry and Day 2003). Interestingly, in the pipefish (Syngnathidae), the positive relationship between offspring size and female size characterized pouch-brooding species but not ventral-brooding species (Braga Goncalves et al. 2011). In isopods, the positive correlation between female size and offspring size was also demonstrated on the interspecific level (Sutton et al. 1984). Different phenomena have been invoked to understand why larger females might produce larger offspring, including competition between siblings (Parker and Begon 1986), unequal benefits from increased fecundity in small vs large females (McGinley 1989), varying efficiency of resource acquisition from parents (Sakai and Harada 2001), increased parental mortality during reproduction (Kindsvater and Otto 2014), and an increased capacity of larger females to meet the overhead costs of reproduction (Filin 2015). With different degrees of relevance, each of these phenomena might apply to isopods. Nevertheless, here we consider that in live-bearing organisms such as isopods, the survival of offspring during brooding is tightly linked to the survival of the parent, a concept that has helped explain the evolution of indeterminate growth pattern in isopods. According to the life history model of Jørgensen et al. (2011), this tight association promotes increased investment in individual offspring by larger females if larger females have improved survival compared to smaller females. If the development of larger offspring requires longer brooding and if brooding is costly, then the production of larger offspring should be more beneficial to larger females because brooding is relatively less costly for them. Importantly, this scenario can help to rationalise the complex pattern found in our data on *P.scaber*. It is suggestive that larger females produced larger offspring only if we considered small broods. We can expect that a small brood (several offspring in our case) is relatively more costly for small females than for large females, which have much higher reproductive potential (more than 100 offspring in our case). If the cost of brooding corresponds to the risk of mortality, then larger brooding females with small broods should suffer relatively lower costs, which should select them for increased investment in individual offspring. Certainly, before drawing firm conclusions regarding this phenomenon, future studies should better identify how the costs of marsupial brooding change with clutch size and female size.

## Conclusions

Based on the integrated findings reported here, we can attempt to form conclusions about the most common patterns in the size dependence of isopod reproduction and the significance of these patterns for understanding the evolution of isopod life histories. In nearly all the studied species, we found a strong size dependence of female reproductive capacity. Such a dependence is important for explaining the evolution of an indeterminate growth strategy in many species of isopods. Data from nearly half of the isopod species revealed a negative relationship between offspring size and offspring number and a positive relationship between mother size and offspring size. Importantly, our case study of *P.scaber* suggests that the emergence of each pattern is context-dependent: a positive effect of female size on offspring size was observed only in smaller broods, and a negative relationship between clutch size and offspring size was observed only for larger females. We propose that these patterns be viewed as different elements of a single phenomenon: a lifetime strategy of investment in growth, reproduction and the parental care provided to single offspring that is shaped by selective conditions. The key message of this study is that to gain a better understanding of this strategy in isopods, we must consider the effects of marsupial brooding, especially its costs and the linkage between the survival of mothers and that of their offspring. We hope that our synthesis of theoretical ideas and data on isopods will increase the intersection of life history theory and empirical research in isopods and that this work will stimulate further theory development and lead to an improved understanding of the ecology and evolution of isopods.

## Supplementary materials

Supplementary material 1 Table S1 Data type: species data Explanation note: Results of the literature search for reports of at least one of the following relationships in isopods: clutch size vs female size, offspring size vs female size, offspring size vs clutch size. This is an open access article distributed under the terms of the Creative Commons Attribution License (CC BY 4.0), which permits unrestricted use, distribution, and reproduction in any medium, provided the original author and source are credited.Andrzej Antoł, Marcin Czarnoleski
