# Supplementary material for: Size dependence of offspring production in isopods: a synthesis
Source: Zookeys. 2018 Dec 3;(801):337–57. doi: 10.3897/zookeys.801.23677 (PMC6288269; doi:10.3897/zookeys.801.23677)
Supplement: Supplementary material 1 — Table S1 [file zookeys-801-337-s001.docx]

Supplementary material

Table 1

Results of the literature search for reports of at least one of the following relationships in isopods: clutch size vs female size, offspring size vs female size, offspring size vs clutch size. The “Habitat” column labels each species according to its typical lifestyle: aquatic or terrestrial, or aquatic/terrestrial if it occupies an intertidal zone. The “Effect” column indicates whether a given relation was positively/negatively significant (+ or -) or nonsignificant (NS). If available, the values of the correlation coefficients (r) are reported next to each relation. The offspring size vs clutch size relationship was studied in two ways, both with and without simultaneous consideration of the effect of female size. The analyses that considered female size focused on a trade-off between allocation to clutch size and to offspring size. To differentiate between these two non-equivalent results, the results obtained with consideration of the effects of female size are marked by asterisks (*). The trade-off present at the interpopulation level is marked with two asterisks (**). The “Size measures” column shows what size measures were used to study the relationship between clutch size and female size: e.n. – egg/embryo number, m.n. – manca number, b.l. – body length, b.m. – body mass (w.e. – with eggs), b.w. – body width, c.l. – cephalic length, c.w. – cephalothorax width, h.w. – head width, b.a. – body area, log – logarithmic transformation of data, cube – cubic transformation of data.

|  |  | **Clutch size vs female size** | | | | **Female size vs offspring size** | | **Clutch size vs offspring size** | |  |  |
| --- | --- | --- | --- | --- | --- | --- | --- | --- | --- | --- | --- |
| **Species** | **Habitat** | **Effect** | **r** | **Size measures** | | **Effect** | **r** | **Effect** | **r** | **Location** | **Ref.** |
| *Aega antarctica* | aquatic | + |  | e.n.  b.l. | |  |  |  |  | Weddell Sea | [65] |
| *Agabiformius lentus* | terrestrial | + | 0.89 | e.n.  b.l. | |  |  |  |  | Morocco | [4] |
| *Agabiformius lentus* | terrestrial | + | 0.7 | m.n.  b.l. | |  |  |  |  | Morocco | [4] |
| *Agabiformius obtusus* | terrestrial | + | 0.55 | m.n.  b.m. | |  |  |  |  | Israel | [51] |
| *Agabiformius obtusus* | terrestrial | + |  | e.n.  b.m. | |  |  |  |  | Israel | [55] |
| *Anilocra apogonae* | aquatic | + | 0.84 | m.n.  b.l. | |  |  |  |  | Australia | [70] |
| *Aphiloscia maculicornis* | terrestrial |  |  |  | | NS |  |  |  | South Africa | [5] |
| *Aphiloscia victoriana* | terrestrial |  |  |  | | NS |  |  |  | South Africa | [5] |
| *Aphiloscia vilis* | terrestrial |  |  |  | | NS | -0.1 | NS | 0.1 | South Africa | [5] |
| *Aphiloscia vilis* | terrestrial |  |  |  | | NS | -0.1 | NS | -0.2 | South Africa | [5] |
| *Aphiloscia vilis* | terrestrial | + | 0.45 | m.n.  b.m. | |  |  |  |  | South Africa | [10] |
| *Aphiloscia vilis* | terrestrial | NS | 0.32 | m.n.  b.m. | |  |  |  |  | South Africa | [10] |
| *Armadillidium granulatum* | terrestrial | + | 0.89 | e.n.  b.l. | |  |  |  |  | Morocco | [4] |
| *Armadillidium granulatum* | terrestrial | + | 0.89 | m.n.  b.l. | |  |  |  |  | Morocco | [4] |
| *Armadillidium nasatum* | terrestrial | + | 0.89 | e.n.  c.l. | |  |  |  |  | Uruguay | [6] |
| *Armadillidium nasatum* | terrestrial | + | 0.88 | log e.n.  log b.m | | + | 0.5 |  |  | Pennsylvania | [7] |
| *Armadillidium nasatum* | terrestrial | + | 0.85 | log e.n.  log b.m | | NS | 0.19 |  |  | Pennsylvania | [7] |
| *Armadillidium pelagicum* | terrestrial | + | 0.7 | e.n.  b.m. | |  |  |  |  | Tunisia | [9] |
| *Armadillidium vulgare* | terrestrial | + |  | m.n.  b.m. | | NS |  | -* |  | Texas | [11] |
| *Armadillidium vulgare* | terrestrial | + | 0.95 | e.n.  b.l. | |  |  |  |  | Japan | [12] |
| *Armadillidium vulgare* | terrestrial | + | 0.84 | log e.n.  log b.m | | + | 0.41 |  |  | Pennsylvania | [7] |
| *Armadillidium vulgare* | terrestrial | + | 0.94 | log e.n  log b.m | | NS | -0.1 |  |  | Pennsylvania | [7] |
| *Armadillidium vulgare* | terrestrial | + |  | e.n.  b.m. | | NS |  | -* | -0.66 | California | [13] |
| *Armadillidium vulgare* | terrestrial | + | 0.85 | m.n.  b.l. | |  |  |  |  | California | [14] |
| *Armadillidium vulgare* | terrestrial | + | + | e.n.  b.l. | |  |  |  |  | Texas | [15] |
| *Armadillidium vulgare* | terrestrial | + | 0.98 | e.n.  b.l. | |  |  |  |  | Texas | [15] |
| *Armadillidium vulgare* | terrestrial | + | 0.98 | e.n.  b.l. | |  |  |  |  | Texas | [15] |
| *Armadillidium vulgare* | terrestrial | + | 1 | m.n.  b.l. | |  |  |  |  | Texas | [15] |
| *Armadillidium vulgare* | terrestrial | + | 1 | m.n.  b.l. | |  |  |  |  | Texas | [15] |
| *Armadillidium vulgare* | terrestrial | + | 1 | m.n.  b.l. | |  |  |  |  | Texas | [15] |
| *Armadillidium vulgare* | terrestrial | + | 0.71 | e.n.  c.l. | |  |  |  |  | Uruguay | [6] |
| *Armadillidium vulgare* | terrestrial | + | 0.69 | e.n.  c.l. | |  |  |  |  | Uruguay | [6] |
| *Armadillidium vulgare* | terrestrial | + | 0.72 | m.n.  c.l. | |  |  |  |  | Uruguay | [6] |
| *Armadillidium vulgare* | terrestrial | + |  | e.n.  h.w. | |  |  |  |  | Japan | [64] |
| *Armadillo officinalis* | terrestrial | + | 0.66 | m.n.  b.m. | |  |  |  |  | Libya | [8] |
| *Armadillo officinalis* | terrestrial | + | 0.87 | m.n.  b.m. | |  |  |  |  | Israel | [51] |
| *Asellus aquaticus* | aquatic | + | 0.96 | e.n.  b.l. | |  |  |  |  | Great Britain | [1] |
| *Asellus meridianus* | aquatic | + | 0.93 | e.n.  b.l. | |  |  |  |  | Great Britain | [1] |
| *Atlantoscia floridana* | terrestrial | + | 0.56 | log m.n.  log c.w. | |  |  |  |  | Brazil | [2] |
| *Atlantoscia floridana* | terrestrial | + | 0.77 | e.n.  c.w. | |  |  |  |  | Brazil | [3] |
| *Atlantoscia floridana* | terrestrial | + | 0.71 | e.n.  c.w. | |  |  |  |  | Brazil | [3] |
| *Atlantoscia floridana* | terrestrial | + | 0.64 | m.n.  c.w. | |  |  |  |  | Brazil | [3] |
| *Balloniscus glaber* | terrestrial | + | 0.62 | log m.n.  log c.w. | |  |  |  |  | Brazil | [2] |
| *Balloniscus sellowii* | terrestrial | + | 0.66 | log m.n.  log c.w. | |  |  |  |  | Brazil | [2] |
| *Benthana cairensis* | terrestrial | + | 0.62 | e.n.  b.w. | |  |  |  |  | Brazil | [16] |
| *Benthana cairensis* | terrestrial | NS |  | m.n.  b.w. | |  |  |  |  | Brazil | [16] |
| *Bethalus pretoriensis* | terrestrial |  |  |  | | + | 0.58 | + | 0.5 | South Africa | [5] |
| *Bethalus pretoriensis* | terrestrial | + | 0.87 | m.n.  b.m. | |  |  |  |  | South Africa | [10] |
| *Burmoniscus ocellatus* | terrestrial | + | 0.88 | e.n.  b.m.w.e. | |  |  |  |  | Hong Kong | [17] |
| *Caecidotea racovitzai* | aquatic | + | 0.45 | log e.n.  log b.m | | NS | 0.33 |  |  | Pennsylvania | [7] |
| *Caecidotea racovitzai* | aquatic | + | 0.77 | log e.n.  log b.m | | NS | 0 |  |  | Pennsylvania | [7] |
| *Cirolana harfordi* | aquatic | + |  | e.n.  b.l. | |  |  |  |  | California | [21] |
| *Cirolana imposita* | aquatic/terrestrial | + | 0.82 | e.n.  b.l. | |  |  |  |  | South Africa | [22] |
| *Clypeoniscus hanseni* | aquatic | + |  | log e.n.  log b.l. | |  |  |  |  | Great Britain | [20] |
| *Cyathura carinata* | aquatic | + | 0.52 | e.n.  c.l. | |  |  |  |  | Portugal | [18] |
| *Cyathura carinata* | aquatic | + | 0.71 | e.n.  c.l. | |  |  |  |  | Portugal | [18] |
| *Cyathura carinata* | aquatic | NS |  | m.n.  c.l. | |  |  |  |  | Portugal | [18] |
| *Cyathura carinata* | aquatic | + | 0.63 | e.n.  b.l. | |  |  |  |  | Sweden | [67] |
| *Cylisticus convexus* | terrestrial | + |  | e.n.  h.w. | |  |  |  |  | Maryland | [19] |
| *Dynamene bidentata* | aquatic/terrestrial | + |  | e.n.  b.a. | |  |  |  |  | Great Britain | [23] |
| *Eurydice affinis* | aquatic | + |  | e.n.  b.l. | |  |  |  |  | Great Britain | [24] |
| *Eurydice pulchra* | aquatic | + |  | e.n.  b.l. | |  |  |  |  | Great Britain | [24] |
| *Excirolana braziliensis* | aquatic/terrestrial | + | 0.66 | e.n.  b.l. | |  |  |  |  | Brazil | [69] |
| *Excirolana chiltoni* | aquatic/terrestrial | + |  | e.n.  b.l. | |  |  |  |  | California | [25] |
| *Excirolana chiltoni* | aquatic/terrestrial | + |  | e.n.  b.l. | |  |  |  |  | California | [25] |
| *Formosillo rafaelei* | terrestrial | + | 0.85 | e.n.  b.m.w.e. | |  |  |  |  | Hong Kong | [17] |
| *Hemileipistus klugii* | terrestrial | + | 0.71 | e.n.  c.w. | |  |  |  |  | Iran | [26] |
| *Hemileipistus reamuri* | terrestrial | + | 0.5 | m.n.  b.m. | |  |  |  |  | Israel | [51] |
| *Idotea balthica basteri* | aquatic | + | 0.89 | e.n.  b.l. | |  |  |  |  | Tunisia | [27] |
| *Idotea balthica basteri* | aquatic | + | 0.92 | m.n.  b.l. | |  |  |  |  | Tunisia | [27] |
| *Idotea balthica basteri* | aquatic | + | 0.8 | e.n.  b.l. | |  |  |  |  | Tunisia | [28] |
| *Idotea balthica basteri* | aquatic | + | 0.91 | m.n.  b.l. | |  |  |  |  | Tunisia | [28] |
| *Idotea balthica* | aquatic | + |  | log e.n.  log b.l | | + |  |  |  | Denmark | [29] |
| *Idotea balthica* | aquatic | + |  | log e.n.  log b.l | | + |  |  |  | Denmark | [29] |
| *Idotea balthica* | aquatic | + | 0.81 | e.n.  b.l. | |  |  |  |  | Nova Scotia | [30] |
| *Idotea balthica* | aquatic | + | 0.64 | e.n.  b.m. | |  |  |  |  | Finland | [31] |
| *Idotea balthica* | aquatic | + | 0.67 | e.n.  b.l. | | NS |  |  |  | Massachusetts | [59] |
| *Idotea balthica* | aquatic | + | 0.62 | e.n.  b.l. | | NS |  |  |  | Massachusetts | [59] |
| *Idotea balthica* | aquatic | + | 0.58 | e.n.  b.l. | | NS |  |  |  | Virginia | [59] |
| *Idotea chelipes* | aquatic | + |  | log e.n.  log b.l | | + |  |  |  | Denmark | [29] |
| *Idotea chelipes* | aquatic | + |  | log e.n.  log b.l | | NS |  |  |  | Denmark | [29] |
| *Idotea granulosa* | aquatic | + | 0.71 | e.n.  b.l. | |  |  |  |  | Iceland | [32] |
| *Idotea granulosa* | aquatic | + | 0.62 | e.n.  b.l. | |  |  |  |  | Iceland | [32] |
| *Idotea granulosa* | aquatic | + | 0.75 | e.n.  b.l. | |  |  |  |  | Iceland | [32] |
| *Idotea granulosa* | aquatic | + | 0.62 | e.n.  b.l. | |  |  |  |  | Iceland | [32] |
| *Idotea granulosa* | aquatic | + | 0.74 | e.n.  b.l. | |  |  |  |  | Iceland | [32] |
| *Idotea granulosa* | aquatic | + | 0.44 | e.n.  b.l. | |  |  |  |  | Iceland | [32] |
| *Idotea granulosa* | aquatic | + | 0.58 | e.n.  b.l. | |  |  |  |  | Iceland | [32] |
| *Idotea granulosa* | aquatic | + | 0.98 | e.n.  b.l. | |  |  |  |  | Irish Sea | [33] |
| *Idotea granulosa* | aquatic | + | 0.97 | e.n.  b.l. | |  |  |  |  | Irish Sea | [33] |
| *Idotea granulosa* | aquatic | + | 0.94 | e.n.  b.l. | |  |  |  |  | Irish Sea | [33] |
| *Idotea pelagica* | aquatic | + | 0.39 | e.n.  b.l. | |  |  |  |  | Iceland | [32] |
| *Idotea pelagica* | aquatic | + | 0.83 | e.n.  b.l. | |  |  |  |  | Iceland | [32] |
| *Idotea pelagica* | aquatic | + | 0.56 | e.n.  b.l. | |  |  |  |  | Iceland | [32] |
| *Idotea pelagica* | aquatic | NS |  | e.n.  b.l. | |  |  |  |  | Iceland | [32] |
| *Idotea pelagica* | aquatic | + |  | log e.n.  log b.l | |  |  |  |  | Great Britain | [20] |
| *Jaera albifrons* | aquatic | + |  | e.n.  b.l. | |  |  |  |  | Great Britain | [34] |
| *Jaera frosmani* | aquatic | + |  | e.n.  b.l. | |  |  |  |  | Great Britain | [34] |
| *Jaera ischiosetosa* | aquatic | + |  | e.n.  b.l. | |  |  |  |  | Great Britain | [34] |
| *Jaera praehirsuta* | aquatic | + |  | e.n.  b.l. | |  |  |  |  | Great Britain | [34] |
| *Janaira gracilis* | aquatic | + | 0.78 | cube e.n.  b.l. | | + | 0.57 |  |  | Brazil | [35] |
| *Ligia cinerascens* | aquatic/terrestrial | + | 0.62 | cube e.n.  b.l. | |  |  |  |  | Japan | [36] |
| *Ligia oceanica* | aquatic/terrestrial | + |  | e.n.  b.m. | | + |  | -* | -0.7 | Great Britain | [37] |
| *Ligia oceanica* | aquatic/terrestrial | + |  | e.n.  b.m. | | + |  | NS* | 0 | Great Britain | [37] |
| *Ligia oceanica* | aquatic/terrestrial | + |  | e.n.  b.m. | | + |  | -* | -0.5 | Great Britain | [37] |
| *Ligia oceanica* | aquatic/terrestrial | + |  | e.n.  b.m. | | + |  | -* | -0.4 | Great Britain | [37] |
| *Ligia pallasii* | aquatic/terrestrial | NS |  | e.n.  b.l. | |  |  |  |  | Canada | [68] |
| *Ligidum japonicum* | terrestrial | + | 0.74 | e.n.  b.l. | |  |  |  |  | Japan | [12] |
| *Lirceus brachyurus* | aquatic | + | 0.48 | log e.n.  log b.m | | NS | -0.5 |  |  | Pennsylvania | [7] |
| *Lirceus brachyurus* | aquatic | + | 0.82 | log e.n.  log b.m | | NS | -0.3 |  |  | Pennsylvania | [7] |
| *Mesidotea entomon* | aquatic | + | 0.8 | e.n.  b.l. | |  |  |  |  | Bothnian Sea | [66] |
| *Mothocya epimerica* | aquatic | + | 0.82 | e.n.  b.l. | |  |  |  |  | Greece | [71] |
| *Mothocya epimerica* | aquatic | + | 0.79 | e.n.  b.m. | |  |  |  |  | Greece | [71] |
| *Mongoloniscus koreanus* | terrestrial | + |  | e.n.  h.w. | |  |  |  |  | Japan | [64] |
| *Oniscus asellus* | terrestrial | + |  | e.n.  b.w./b.l. | |  |  |  |  | Great Britain | [38] |
| *Oniscus asellus* | terrestrial | + | 0.94 | e.n.  cube b. l. | |  |  |  |  | France | [61] |
| *Oniscus asellus* | terrestrial | + | 0.91 | e.n.  cube b. l. | |  |  |  |  | France | [61] |
| *Oniscus asellus* | terrestrial | + | 0.69 | e.n.  cube b. l. | |  |  |  |  | France | [61] |
| *Orodillo maculatus* | terrestrial | + | 0.74 | e.n.  b.m.w.e. | |  |  |  |  | Hong Kong | [17] |
| *Philoscia muscorum* | terrestrial | + | 0.93 | e.n.  h.w. | |  |  |  |  | Great Britain | [41] |
| *Philoscia muscorum* | terrestrial | + | 0.79 | e.n.  h.w. | |  |  |  |  | Great Britain | [41] |
| *Philoscia muscorum* | terrestrial | + |  | e.n.  h.w. | |  |  |  |  | Great Britain | [42] |
| *Porcellio albinus* | terrestrial | + | 0.66 | e.n.  b.l. | | NS |  |  |  | Tunisia | [63] |
| *Porcellio barroisi* | terrestrial | + |  | e.n.  b.m. | |  |  |  |  | Israel | [54] |
| *Porcellio buddelundi* | terrestrial | + | 0.95 | e.n.  b.m. | |  |  |  |  | Tunisia | [39] |
| *Porcellio buddelundi* | terrestrial | + | 0.84 | e.n.  b.m. | |  |  |  |  | Tunisia | [39] |
| *Porcellio dalensis* | terrestrial | + | 0.95 | e.n.  b.l. | |  |  |  |  | Morocco | [4] |
| *Porcellio dalensis* | terrestrial | + | 0.95 | m.n.  b.l. | |  |  |  |  | Morocco | [4] |
| *Porcellio ficulneus* | terrestrial | NS | 0.25 | m.n.  b.m. | |  |  |  |  | Israel | [51] |
| *Porcellio laevis* | terrestrial | + | 0.62 | e.n.  b.l. | |  |  |  |  | Chile | [57] |
| *Porcellio laevis* | terrestrial | + | 0.86 | e.n.  b.l. | |  |  |  |  | Chile | [57] |
| *Porcellio laevis* | terrestrial | + | 0.94 | e.n.  b.m.w.e. | |  |  |  |  | Chile | [56] |
| *Porcellio laevis* | terrestrial | + | 0.95 | e.n.  b.l. | |  |  |  |  | Morocco | [4] |
| *Porcellio laevis* | terrestrial | + | 0.95 | m.n.  b.l. | |  |  |  |  | Morocco | [4] |
| *Porcellio laevis* | terrestrial | NS | 0.08 | m.n.  b.m. | |  |  |  |  | Israel | [51] |
| *Porcellio lamellatus* | terrestrial | + |  | e.n.  b.l. | |  |  |  |  | Tunisia | [58] |
| *Porcellio lamellatus* | terrestrial | + |  | e.n.  b.l. | |  |  |  |  | Tunisia | [58] |
| *Porcellio olivieri* | terrestrial | + |  | e.n.  b.m. | |  |  |  |  | Israel | [55] |
| *Porcellio scaber* | terrestrial | + | 0.83 | | log m.n.  log b.m. | NS | 0.27 | NS* | 0.12 | Poland | this  study |
| *Porcellio scaber* | terrestrial | + | 0.83 | log m.n.  log b.m. | | + | 0.6 | -* | -0.54 | Poland | this study |
| *Porcellio scaber* | terrestrial | + | 0.83 | log m.n.  log b.m. | | NS | 0.1 | NS | 0.02 | Poland | this study |
| *Porcellio scaber* | terrestrial | + |  | m.n.  b.m. | |  |  |  |  | Poland | [60] |
| *Porcellio scaber* | terrestrial | + | 0.82 | e.n.  b.l. | |  |  |  |  | Libya | [44] |
| *Porcellio scaber* | terrestrial | + | 0.69 | e.n.  b.l. | |  |  |  |  | Japan | [12] |
| *Porcellio scaber* | terrestrial | + | 0.75 | log e.n.  log b.m | | NS | 0 |  |  | Pennsylvania | [7] |
| *Porcellio siculoccidentalis* | terrestrial | + | 0.92 | e.n.  b.l. | |  |  |  |  | Italy | [45] |
| *Porcellio siculoccidentalis* | terrestrial | + | 0.97 | e.n.  b.l. | |  |  |  |  | Italy | [45] |
| *Porcellio siculoccidentalis* | terrestrial | + | 0.92 | m.n.  c.l. | |  |  |  |  | Italy | [45] |
| *Porcellio variabilis* | terrestrial | + | 0.62 | e.n.  b.l. | |  |  |  |  | Tunisia | [46] |
| *Porcellio variabilis* | terrestrial | + | 0.82 | e.n.  b.l. | |  |  |  |  | Tunisia | [46] |
| *Porcellionides sexfasciatus* | terrestrial | + | 0.95 | e.n.  b.l. | |  |  |  |  | Morocco | [4] |
| *Porcellionides sexfasciatus* | terrestrial | + | 0.95 | m.n.  b.l. | |  |  |  |  | Morocco | [4] |
| *Porcellionides pruinosus* | terrestrial | + | 0.25 | m.n.  b.m. | |  |  |  |  | Zimbabwe | [10] |
| *Porcellionides pruinosus* | terrestrial | NS | 0.52 | m.n.  b.m. | |  |  |  |  | South Africa | [10] |
| *Porcellionides pruinosus* | terrestrial | + | 0.85 | e.n.  b.l. | |  |  |  |  | Tunisia | [43] |
| *Porcellionides pruinosus* | terrestrial | + | 0.89 | m.n.  b.l. | |  |  |  |  | Tunisia | [43] |
| *Porcellionides pruinosus* | terrestrial | + | 0.95 | e.n.  b.l. | |  |  |  |  | Morocco | [4] |
| *Porcellionides pruinosus* | terrestrial | + | 0.95 | m.n.  b.l. | |  |  |  |  | Morocco | [4] |
| *Porcellionides pruinosus* | terrestrial |  |  |  | | + | 0.28 | NS | 0.1 | Zimbabwe | [5] |
| *Porcellionides pruinosus* | terrestrial |  |  |  | | NS | 0.09 | NS | -0.2 | Botswana | [5] |
| *Porcellionides pruinosus* | terrestrial | + |  | m.n.  b.m. | | NS |  |  |  | Zimbabwe | [62] |
| *Porcellium colicola* | terrestrial | + | 0.82 | e.n.  b.l. | |  |  |  |  | Romania | [40] |
| *Schizidium tiberianum* | terrestrial | + | 0.77 | m.n.  b.m. | |  |  |  |  | Israel | [51] |
| *Serolis cornuta* | aquatic | + |  | e.n.  b.l. | |  |  |  |  | Antarctic | [47] |
| *Serolis cornuta* | aquatic | + |  | e.n.  b.w. | | NS |  | -** |  | Weddell Sea | [48] |
| *Serolis cornuta* | aquatic | + |  | e.n.  b.w. | | NS |  | -** |  | Weddell Sea | [48] |
| *Serolis cornuta* | aquatic |  |  |  | | + |  | -** |  | Signy Island | [48] |
| *Serolis cornuta* | aquatic | + | 0.73 | e.n.  b.w. | |  |  |  |  | Antarctic | [49] |
| *Serolis pagenstecheri* | aquatic | + | 0.81 | e.n.  b.w. | |  |  |  |  | Antarctic | [49] |
| *Serolis polita* | aquatic | + | 0.41 | e.n.  b.w. | |  |  |  |  | Antarctic | [49] |
| *Serolis septemcarinata* | aquatic | + | 0.67 | e.n.  b.w. | |  |  |  |  | Antarctic | [49] |
| *Soteriscus gaditanus* | terrestrial | + | 0.89 | e.n.  b.l. | |  |  |  |  | Morocco | [4] |
| *Soteriscus gaditanus* | terrestrial | + | 0.89 | m.n.  b.l. | |  |  |  |  | Morocco | [4] |
| *Sphaeroma serratum* | aquatic | NS | 0.02 | e.n.  b.l. | |  |  |  |  | Tunisia | [50] |
| *Trachelipus arcuatus* | terrestrial | + | 0.85 | e.n.  b.l. | |  |  |  |  | Romania | [40] |
| *Trachelipus nodulosus* | terrestrial | + |  |  | |  |  |  |  | Hungary | [53] |
| *Trachelipus rathkii* | terrestrial | + |  | e.n.  h.w. | |  |  |  |  | Maryland | [19] |
| *Trachelipus rathkii* | terrestrial | + | 0.87 | log e.n.  log b.m | | NS | 0.06 |  |  | Pennsylvania | [7] |
| *Trachelipus rathkii* | terrestrial | + | 0.73 | log e.n.  log b.m | | - | -0.5 |  |  | Pennsylvania | [7] |
| *Trachelipus rathkii* | terrestrial | + | 0.87 | log e.n.  log b.m | | NS | 0.06 |  |  | Pennsylvania | [7] |
| *Trachelipus rathkii* | terrestrial | + | 0.73 | log e.n.  log b.m | | - | -0.5 |  |  | Pennsylvania | [7] |
| *Trichoniscus pusillus* | terrestrial | + | 0.63 | log e.n.  log b.m | | NS | 0.05 |  |  | Pennsylvania | [7] |
| *Trichoniscus pusillus* | terrestrial | + |  | e.n.  h.w. | |  |  |  |  | Great Britain | [42] |

[1] Steel 1961, [2] Quadros et al. 2007, [3] Araujo and Bond-Buckup 2005, [4] Achouri et al. 2008, [5] Telford and Dangerfield 1995, [6] Waller and Verdi 2016, [7] Glazier 2003, [8] AlJetlawi and Nair 1994, [9] Hamaied and Charfi-Cheikrouha 2004, [10] Dangerfield and Telford 1995, [11] Brody and Lawlor 1984, [12] Saito 1969, [13] Lawlor 1976, [14] Paris and Pitelka 1962, [15] Miller and Cameron 1983, [16] Sokolowicz and Araujo 2013, [17] Ma et al. 1991, [18] Marques et al. 1994, [19] Hornung et al. 2015, [20] Sheader 1977, [21] Johnson 1976, [22] Shafir 1980, [23] Holdich 1968, [24] Jones 1970, [25] Klapow 1970, [26] Kashani et al. 2011, [27] Zaabar et al. 2014, [28] Zaabar et al. 2016, [29] Kroer 1989, [30] Strong and Daborn 1979, [31] Jormalainen et al. 2001, [32] Leifsson 1998, [33] Salemaa 1986, [34] Jones 1971, [35] Moreira and Pires 1977, [36] Furota and Ito 1999, [37] Willows 1987, [38] Phillipson and Watson 1965, [39] Medini-Bouaziz et al. 2017, [40] Ivanov 2011, [41] Sunderland et al. 1976, [42] Sutton 1968, [43] Achouri et al. 2002, [44] Nair 1998, [45] Montesanto el al. 2012, [46] Medini-Bouaziz et al. 2015, [47] Wägele 1987, [48] Clarke and Gore 1992, [49] Luxmoore 1982, [50] Ben Souissi et al. 2015, [51] Warburg 2013, [52] Gonçalves et al. 2005, [53] Hornung 1988, [54] Warburg (1995a), [55] Warburg (1995b), [56] Lardies et al. (2004a), [57] Lardies et al. (2004b), [58] Khemaissia et al. 2016, [59] Manyak-Davis et al. 2013, [60] Horvathova et al. 2017, [61] Rigaud et al. 1999, [62] Dangerfield and Telford (1990), [63] Medini-Bouaziz et al. 2017, [64] Tanaka and Karasawa (2018), [65] Wägele 1990, [66] Leonardsson 1986, [67] Ólafsson and Persson 1986, [68] Carefoot 1973, [69] Fonesca et al. 2000, [70] Fogelman and Grutter 2008, [71] Leonardos and Trilles 2009
